# The utility of SARS‐CoV‐2 nucleocapsid protein in laboratory diagnosis

**DOI:** 10.1002/jcla.24534

**Published:** 2022-06-03

**Authors:** Xinwei Li, Mengyuan Xiong, Qiaoling Deng, Xiaobing Guo, Yirong Li

**Affiliations:** ^1^ Class 11, Grade 2018 Medical School of Zhengzhou University Zhengzhou China; ^2^ Department of Laboratory Medicine, Zhongnan Hospital Wuhan University Wuhan China; ^3^ Department of Laboratory Medicine The First Affiliated Hospital of Zhengzhou University Zhengzhou China

**Keywords:** antigen detection, COVID‐19, laboratory diagnosis, nucleocapsid protein, SARS‐CoV‐2

## Abstract

**Background:**

The Coronavirus Disease 2019 (COVID‐19) is caused by the severe acute respiratory syndrome coronavirus 2 (SARS‐CoV‐2), which has now become a global pandemic owing to its high transmissibility. The SARS‐CoV‐2 nucleocapsid protein tests are playing an important role in screening and diagnosing patients with COVID‐19, and studies about the utility of SARS‐CoV‐2 nucleocapsid protein tests are increasing now.

**Methods:**

In this review, all the relevant original studies were assessed by searching in electronic databases including Scopus, Pubmed, Embase, and Web of Science. “SARS‐CoV‐2”, “COVID‐19”, “nucleocapsid protein”, and “antigen detection” were used as keywords.

**Results:**

In this review, we summarized the utility of SARS‐CoV‐2 nucleocapsid protein in laboratory diagnosis. Among the representative researches, this review analyzed, the sensitivity of SARS‐CoV‐2 nucleocapsid protein detection varies from 13% to 87.9%, while the specificity could almost reach 100% in most studies. As a matter of fact, the sensitivity is around 50% and could be higher or lower due to the influential factors.

**Conclusion:**

It is well suggested that SARS‐CoV‐2 nucleocapsid protein is a convenient method with a short turnaround time of about half an hour, and the presence of N antigen is positively related to viral transmissibility, indicating that SARS‐CoV‐2 N protein immunoassays contribute to finding out those infected people rapidly and segregating them from the uninfected people.

## Graphical abstract

In this review, five immunoassays for detecting the SARS‐CoV‐2 N antigen, including LF‐CGIA, LF‐IFA, ELISA, CLIA, and BAW biosensor‐based immunoassay were introduced. These five assays could be the supplement of RT‐PCR and could aid the policy of “early diagnosis and early treatment”, indicating that SARS‐CoV‐2 N protein immunoassays contribute to finding out those infected people rapidly and segregating them from the uninfected people.

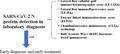

## 1 INTRODUCTION

By the end of December 2019, several unknown pneumonia cases who manifested as respiratory syndromes and fever were found in Wuhan, China.^1^ Subsequently, severe acute respiratory syndrome coronavirus 2 (SARS‐CoV‐2), a novel virus classified as β‐coronavirus genus, was identified in bronchoalveolar lavage fluid and other respiratory samples obtained from patients with unknown pneumonia.^2^ As of March 31, 2022, about 485 million confirmed cases have been reported worldwide with more than 6.1 million deaths.^3^

With the incessant transmission of COVID‐19, SARS‐CoV‐2 has mutated into a variety of variants due to numerous duplications. As a result, some of these variants are highly contagious. How to prevent SARS‐CoV‐2 from mutating and transmitting seems to be a big problem. It is universally acknowledged that there are three basic segments in preventing infectious disease from spreading, which are eliminating the infection sources, cutting off the infection routes, and protecting the susceptible people. Truths are that utilizing a method with accuracy and rapidity could find out those who are infected with COVID‐19 in a short period of time. Therefore, we could separate the infected person in time and successfully prevent the spread of this infectious disease. In a word, it is of great significance to work out the best way to rapidly screen and diagnose patient with COVID‐19.

Real‐time reverse transcriptase polymerase chain reaction (RT‐PCR), antigen detection, and antibody detection are three different assays used to diagnose COVID‐19, and they are, respectively, based on viral nucleic acid detection, viral protein detection, and human antibody detection.^4^ The viral nucleic acid detection by RT‐PCR is considered as the most reliable and widely used technique.^4^, ^5^ However, the nucleic acid test by RT‐PCR is not convenient because it requires a molecular diagnostic laboratory equipped with trained staff and expensive equipment. Besides, another shortcoming of RT‐PCR assay is the long turnaround time which limits the testing scale.^5^ Viral protein detection could be detected up to 1 day ahead of clinical symptoms onset and is easy‐to‐use, inexpensive, and could be applied on a large scale, while the limitation is the low analytical sensitivity.^6^ The benefit of antibody detection is that the device sometimes can be used at home, but positive results could prove the existence of past or current infection, or the person is vaccinated, and cross‐reactivity is unavoidable.^4^, ^7^, ^8^

### 1.1 Genome structure of SARS‐CoV‐2

On January 10, 2020, the first whole genomic sequences of SARS‐CoV‐2 was published on the Virological website. Genomic analysis shows that SARS‐CoV‐2 is comprised of a positive‐sense, single‐stranded RNA genome of around 30 kb and shares 79% genome sequence identity with SARS‐CoV and 87.9% with bat CoV strain bat‐SL‐CoVZC45 and bat‐SL‐CoVZXC.^2^ The 5′‐terminus of the genome contains ORF1a and ORF1b that encode 16 non‐structural proteins (nsps1‐16).^9^ Most of these SARS‐CoV‐2 non‐structural proteins have more than 85% amino acid sequence identity with SARS‐CoV.^10^ The 3′‐terminus of the genome encodes 4 structural proteins including spike (S), envelope (E), membrane (M), and nucleocapsid (N). In addition, ORFs encode eight accessory proteins and are interspersed among these structural genes.^11^ As the virus spreads, they constantly mutate their genetic code to evolve or adapt due to host immunity.^12^ Most mutations in the SARS‐CoV‐2 genome do not affect the function of the virus, but a few mutations of SARS‐CoV‐2 may make the virus easier to spread, affecting how well vaccines could protect people, causing the virus less responsive to treatments for COVID‐19, and/or even leading to the avoidance of SARS‐CoV‐2 detection,^13^, ^14^, ^15^ which makes it difficult to implement the policy of “early diagnosis and early treatment”. As the duration of the outbreak increases, mutations have occurred more frequently, potentially affecting the infectivity and pathogenicity of the virus. Table 1 compiled the commonest mutation types of SARS‐CoV‐2 in genomics, which demonstrates that most of these mutated sites are on structural gene S. However, different from structural gene S, the N gene is less frequently mutated, indicating that the N protein is relatively conserved and have the potential to be an interesting protein for laboratory diagnosis.

**TABLE 1:** Commonest mutation types of SARS‐CoV‐2 in genomics

NO.	Mutation types	Country of origin	First reported time	Mutation located in nucleoprotein	Mutation located in spike protein	Communicability and mortality modification
1	Alpha (B.1.1.7)	United Kingdom	Late 2020/12	S235F	69–70 deletion, 144 deletion, E484k, S494P, N501Y, A570D, D614G, P681H, T716I, S982A, D1118H, K1191N	43% to 82% more transmissible, mortality hazard ratio was 1.64^43^
2	Beta (B.1.351)	South Africa	2020/12/1	T205I	L18F, D80A, D215G, 241‐243deletion, R246I, K417N, E484K, N501Y, D614G, and A701V	Reduced neutralization by monoclonal antibody therapies, convalescent sera, and post‐vaccination sera^43^
3	Gamma (P.1)	Brazil	Early 2021/01	P80R	L18F, T20N, P26S, D138Y, R190S, H655Y, T1027I V1176, K417T, E484K, and N501Y	Reduced neutralization by monoclonal antibody therapies, convalescent sera, and post‐vaccination sera^43^
4	Delta (B.1.617.2)	India	2020/12/1	Not reported	T19R, T95I, (G142D*), 156del, 157del, R158G, (A222V*), (W258L*), (K417N*) L452R, T478K, D614G, P681R, and D950N	More transmissible, convalescent sera, and post‐vaccination sera^38^
5	Epsilon (B.1.427 and B.1.429)	United States	2020/6/1	Not reported	B.1.427: L452R and D614G; B.1.429: S13I, W152C, L452R, and D614G	More transmissible, reduced neutralization by monoclonal antibody therapies, convalescent sera, and post‐vaccination sera^43^
6	Zeta (P.2)	Brazil	2020/4/1	A119S, R203K, G204R, M234I, and R81C	L18F, T20N, P26S, F157L, E484K, D614G, S929I, and V1176F	Reduced neutralization by monoclonal antibody therapies, convalescent sera, and post‐vaccination sera^43^
7	Eta (B.1.525)	United States	2020/11/1	A12G and T205I	A67V, Δ69/70, Δ144, E484K, D614G, Q677H, and F888L	Reduced neutralization by monoclonal antibody therapies^43^
8	Iota (B.1.526)	United States	Not reported	Not reported	(L5F*), T95I, D253G, (S477N*), (E484K*), D614G, and (A701V*)	Reduced neutralization by monoclonal antibody therapies, convalescent sera, and post‐vaccination sera^43^
9	Theta (P.3) also called GR/1092 K.V1	Philippines and Japan	2021/2/1	Not reported	141–143 deletion E484K, N501Y, and P681H	Not reported
10	Kappa (B.1.617.1)	India	2021/12/1	Not reported	(T95I), G142D, E154K, L452R, E484Q, D614G, P681R, and Q1071H	Reduced neutralization by monoclonal antibody therapies, convalescent sera, and post‐vaccination sera^43^
11	Lambda (C.37)	Peru	2021/6/1	Not reported	Deletion Δ246‐252	Not reported
12	Omicron (B.1.1.529)	South Africa	2021/11/9	Three deletions at E31‐, R32‐, and S33‐, and substitutions at P13L, R203K, and G204R	H69‐, V70‐, G142‐, V143, Y144‐, N211‐ of which 69/70 deletions. Substitutions are A67V, T95I, Y145D, G339D, S371L, S373P, S375F, K417N, N440K, G446S, S477N, T478K, E484A, Q493R, G496S, Q498R, N501Y, Y505H, T547K, D614G, H655Y, N679K, P681H, N764K, D796Y, N856K, Q954H, N969K, and L981F	Omicron variant is involved in infections with recovered individuals May have a greater potential to escape prior immunity than the previous delta variant Vaccines have neutralization capacity reduction against omicron^44^

### 1.2 Structural and functional analysis of SARS‐CoV‐2 N protein

SARS‐CoV‐2 N protein contains 419 amino acids, and is originated from a 1260 nucleotide length N gene after transcription and translation.^16^ Sequence alignment of N protein indicates that the SARS‐CoV‐2 N protein closely resembles the SARS‐CoV N protein rather than other human coronavirus N proteins. SARS‐CoV‐2 N protein consists of two structural domains named as N‐terminal domain (NTD) and C‐terminal domain (CTD), which are separated by a disordered linker and flanked on both termini by disordered tails.^17^ The NTD, primarily responsible for RNA‐binding, can be divided into three regions: a protruded basic finger, a basic palm, and an acidic wrist.^9^ The CTD may function as a bridge in the formation of N protein dimer because it has been proved that CTD‐CTD interaction could be found in the solution. It has been suggested that these two domains are required to bind to viral genome RNA, and then contribute to packing it into ~100 nm particles.^18^, ^19^ The disordered linker between NTD and CTD domains has a S‐rich (SR) region, and could be phosphorylated at multiple sites in vitro by SRPK1, so that the N protein will be recruited to stress granules.^17^ Apart from the functions mentioned above, other functions of N protein include binding with non‐specific dsDNA probably by electrostatic interaction, entering the host cell, and forming the ribonucleoprotein core.^18^

## 2 UTILITY OF SARS‐CoV‐2 N PROTEIN IN LABORATORY DIAGNOSIS

Until late March 2022, about 48 N antigen diagnostic test kits for SARS‐CoV‐2 have been developed and acquired emergency use authorization from US food and drug administration (FDA).^20^ Table 2 listed all authorized kits for emergency use from US FDA.^20^ The most commonly used method in emergency use authorization kits for testing SARS‐CoV‐2 N antigen is lateral flow colloidal gold immunochromatographic assay (LF‐CGIA), following by lateral flow immunofluorescence assay (LF‐IFA) and chemiluminescence immunoassay (CLIA).

**TABLE 2:** Authorized kits for testing SARS‐CoV‐2 N antigen from U.S. FDA

NO.	Entity	Name	Attribute	Sample collection	Sensitivity (PPA)	Specificity (NPA)	Limit of detection	Cross reactivity
1	Quidel Corporation	Sofia 2 Flu + SARS Antigen FIA	LF‐IFA	Nasal swab, nasopharyngeal swab	95.2%	100%	91.7 TCID_50_/ml	None
2	Celltrion USA, Inc.	Sampinute COVID‐19 Antigen MIA	MESIA	Nasopharyngeal swab	94.4%	100%	1.2 × 10^2^ TCID_50_/ml	None
3	Luminostics, Inc.	Clip COVID Rapid Antigen Test	LF‐IFA	Nasal swab	96.9%	100%	0.88 × 10^2^ TCID_50_/ml	Determining to be cross reactive to SARS‐CoV
4	Princeton BioMeditech Corp.	Status COVID‐19/Flu	LF‐CGIA	Nasopharyngeal swab	93.9%	100%	2.7 × 10^3^ TCID_50_/ml	Cannot rule out the cross reactivity with MERS‐CoV and human coronavirus HKU1
5	Ellume Limited	Ellume COVID‐19 Home Test	LF‐IFA	Nasal swab	95%	97%	10^3.80^ TCID_50_/ml	Likely to have cross reactivity with SARS‐CoV
6	Quidel Corporation	QuickVue At‐Home COVID‐19 Test	LF‐CGIA	Nasal swab	84.8%	99.1%	1.91 × 10^4^ TCID_50_/ml	None
7	Ortho Clinical Diagnostics, Inc.	VITROS Immunodiagnostic Products SARS‐CoV‐2 Antigen Reagent Pack	CLIA	Nasal swab, nasopharyngeal swab	75.4% (nasal) 86.2% (nasopharyngeal)	100% (nasal) 97.7% (nasopharyngeal)	7 transport media types ranging from 5.0 × 10^2^ TCID_50_/ml to 3.0 × 10^3^ TCID_50_/ml	Would be significant cross reactivity with SARS‐CoV
8	Becton, Dickinson and Company (BD)	BD Veritor System for Rapid Detection of SARS‐CoV‐2 & Flu A + B	Chromatographic digital immunoassay	Nasal swab	86.7%	99.5%	2.8 × 10^2^ TCID_50_/ml	None
9	Abbott Diagnostics Scarborough, Inc.	BinaxNOW COVID‐19 Ag 2 Card	LF‐CGIA	Nasal swab	84.6%	98.5%	140.6 TCID_50_/ml	Cannot rule out the cross reactivity with MERS‐CoV and human coronavirus HKU1
10	Abbott Diagnostic Scarborough Inc.	BinaxNOW COVID‐19 Ag Card 2 Home Test	LF‐CGIA	Nasal swab	84.6%	98.5%	140.6 TCID_50_/ml	Cannot rule out the cross reactivity with MERS‐CoV and human coronavirus HKU1
11	Quidel Corporation	QuickVue At‐Home OTC COVID‐19 Test	LF‐CGIA	Nasal swab	83.5%	99.2%	1.91 × 10^4^ tcid_50_/ml	None
12	Abbott Diagnostic Scarborough Inc.	BinaxNOW COVID‐19 Ag Card Home Test	LF‐CGIA	Nasal swab	91.7%	100%	140.6 TCID_50_/ml	Cannot rule out the cross reactivity with MERS‐CoV and human coronavirus HKU1
13	Qorvo Biotechnologies, LLC.	Omnia SARS‐CoV‐2 Antigen Test	BAW	Nasal swab	89.47%	100%	200 TCID_50_/ml	Cannot rule out the cross reactivity with SARS‐CoV and human coronavirus HKU1
14	Becton, Dickinson and Company (BD)	BD Veritor System for Rapid Detection of SARS‐CoV‐2	Chromatographic digital immunoassay	Nasal swab	84%	100%	1.4 × 10^2^ TCID_50_/ml	Cannot rule out the cross reactivity with human coronavirus HKU1
15	LumiraDx UK Ltd.	LumiraDx SARS‐CoV‐2 Ag Test	MIFA	Nasal swab, nasopharyngeal swab	97.6% (nasal) 97.5% (nasopharyngeal)	96.6% (nasal) 97.7% (nasopharyngeal)	32 TCID_50_/ml	Likely to have cross reactivity with SARS‐CoV Cannot rule out the cross reactivity with human coronavirus HKU1
16	Abbott Diagnostic Scarborough Inc.	BinaxNOW COVID‐19 Ag Card	LF‐CGIA	Nasal swab	84.6%	98.5%	140.6 TCID_50_/ml	Cannot rule out the cross reactivity with MERS‐CoV and human coronavirus HKU1
17	Salofa Oy	Sienna‐Clarity COVID‐19 Antigen Rapid Test Cassette	LF‐CGIA	Nasopharyngeal swab	87.5%	98.9%	1.25 × 10^3^ TCID_50_/ml	Cannot rule out the cross reactivity with SARS‐CoV and human coronavirus HKU1
18	OraSure Technologies, Inc.	InteliSwab COVID‐19 Rapid Test Pro	LF‐CGIA	Nasal swab	84%	98%	2.5 × 10^2^ TCID_50_/ml	Cannot rule out the cross reactivity with human coronavirus HKU1
19	OraSure Technologies, Inc.	InteliSwab COVID‐19 Rapid Test	LF‐CGIA	Nasal swab	84%	98%	2.5 × 10^2^ TCID_50_/ml	Cannot rule out the cross reactivity with human coronavirus HKU1
20	OraSure Technologies, Inc.	InteliSwab COVID‐19 Rapid Test Rx	LF‐CGIA	Nasal swab	84%	98%	2.5 × 10^2^ TCID_50_/ml	Cannot rule out the cross reactivity with human coronavirus HKU1
21	Quidel Corporation	Sofia SARS Antigen FIA	LF‐IFA	Nasal swab	96.7%	100%	1.13 × 10^2^ TCID_50_/ml	None
22	Ellume Limited	ellume.lab COVID Antigen Test	LF‐IFA	Nasal swab	81.8%	100%	7.16 × 10^3^ TCID_50_/ml	Cannot rule out the cross reactivity with SARS‐CoV and human coronavirus HKU1
23	DiaSorin, Inc.	LIAISON SARS‐CoV‐2 Ag	CLIA	Nasal swab, nasopharyngeal swab	97.0% (nasal) 96.1% (nasopharyngeal)	100% (nasal) 99.3% (nasopharyngeal)	300 TCID_50_/ml (CLASSIQ swab™) 300 TCID_50_/ml (FLOQ swab™) 575 TCID_50_/ml (FLOQ swab™ minitip)	Suggesting cross reactivity with SARS‐CoV Cannot rule out the cross reactivity with human coronavirus HKU1
24	Access Bio, Inc.	CareStart COVID‐19 Antigen test	LF‐CGIA	Nasal swab, nasopharyngeal swab	87.18% (nasal) 93.75% (nasopharyngeal)	100% (nasal) 99.32% (nasopharyngeal)	8 × 10^2^ TCID_50_ /ml	Cannot rule out the cross reactivity with human coronavirus HKU1
25	Quidel Corporation	QuickVue SARS Antigen Test	LF‐CGIA	Nasal swab	96.6%	99.3%	7.57 × 10^3^ TCID_50_ /ml	None
26	PHASE Scientific International, Ltd.	INDICAID COVID‐19 Rapid Antigen Test	LF‐CGIA	Nasal swab	84.4%	96.3%	2.8 × 10^3^ TCID_50_/ml	Cannot rule out the cross reactivity with SARS‐CoV, Mycobacterium tuberculosis, Pneumocystis jirovecii (PJP), and human coronavirus HKU1
27	QIAGEN GmbH	QIAreach SARS‐CoV‐2 Antigen	Digital lateral flow, fluorescence	Nasal swab, nasopharyngeal swab	85.00% (nasal) 80.65% (nasopharyngeal)	99.05% (nasal) 98.31% (nasopharyngeal)	5.0 × 10^4^ TCID_50_/ml	Exhibiting cross reactivity with SARS‐CoV Cannot rule out the cross reactivity with Pneumocystis jirovecii (PJP) and human coronavirus HKU1
28	Abbott Diagnostic Scarborough Inc.	BinaxNOW COVID‐19 Antigen Self Test	LF‐CGIA	Nasal swab	84.6%	98.5%	140.6 TCID_50_/ml	Cannot rule out the cross reactivity with MERS‐CoV and human coronavirus HKU1
29	Access Bio, Inc.	*CareStart* COVID‐19 Antigen Home Test	LF‐CGIA	Nasal swab	87%	98%	2.8 × 10^3^ TCID_50_/ml	Cannot rule out the cross reactivity with Mycoplasma pneumoniae, Mycobacterium tuberculosis, Pneumocystis jirovecii (PJP), and human coronavirus HKU1
30	Becton, Dickinson and Company (BD)	BD Veritor At‐Home COVID‐19 Test	LF‐CGIA	Nasal swab	84.6%	99.8%	1.87 × 10^2^ TCID_50_/ml	Cannot rule out the cross reactivity with Mycobacterium tuberculosis and human coronavirus HKU1
31	Celltrion USA, Inc.	Celltrion DiaTrust COVID‐19 Ag Rapid Test	LF‐CGIA	Nasopharyngeal swab	93.33%	99.03%	3.2 × 10^1^ TCID_50_/ml	Cross reactivity is highly likely with SARS‐CoV Cannot rule out the cross reactivity with Mycobacterium tuberculosis and human coronavirus HKU1
32	InBios International, Inc.	SCoV‐2 Ag Detect Rapid Test	LF‐CGIA	Nasal swab	86.67%	100.00%	6.3 × 10^3^ TCID_50_/ml	Low probability of cross reactivity with human coronavirus HKU1 Predicting to be cross‐reactive with SARS‐CoV
33	Quanterix Corporation	Simoa SARS‐CoV‐2 N Protein Antigen Test	Paramagnetic microbead‐based immunoassay	Nasal swab, nasopharyngeal swab, saliva	88.6% (nasal) 97.7% (nasopharyngeal) 84.1% (saliva)	100% (nasal) 100% (nasopharyngeal) 98.1% (saliva)	0.29 TCID_50_/ml (nasopharyngeal and nasal swab) 0.16 TCID_50_/ml (saliva)	Cannot rule out the cross reactivity with MERS‐CoV, Mycobacterium tuberculosis, Pneumocystis jirovecii (PJP), and human coronavirus HKU1
34	GenBody Inc.	GenBody COVID‐19 Ag	LF‐CGIA	Nasal swab, nasopharyngeal swab	92.31% (nasal) 91.1% (nasopharyngeal)	99.04% (nasal) 100% (nasopharyngeal)	1.11 × 10^2^ TCID_50_/ml	Cannot rule out the cross reactivity with Mycobacterium tuberculosis, Pneumocystis jirovecii (PJP), and human coronavirus HKU1
35	ANP Technologies, Inc	NIDS COVID‐19 Antigen Rapid Test Kit	LF‐CGIA	Nasal swab	95.1%	97.0%	311 TCID_50_/ml	Cross reactivity with SARS virus
36	Xtrava Health	SPERA COVID‐19 Ag Test	LF‐CGIA	Nasal swab	91.8%	96.9%	1.56 × 10^3^ TCID_50_/ml	Cross reactivity with SARS virus
37	ACON Laboratories, Inc	Flowflex COVID‐19 Antigen Home Test	LF‐CGIA	Nasal swab	93%	100%	2.5 × 10^3^ TCID_50_/ml	Cross reactivity with human coronavirus HKU1 cannot be completely ruled out Cross reactivity with SARS virus
38	Princeton BioMeditech Corp.	Status COVID‐19/Flu A&B	LF‐CGIA	Nasal swab, nasopharyngeal swab	93.8%	100%	2.7 × 10^3^ TCID_50_/ml	Likely to have cross reactivity with SARS‐CoV Cross reactivity with Mycobacterium tuberculosis cannot be ruled out
39	InBios International Inc.	SCoV‐2 Ag Detect Rapid Self‐Test	LF‐CGIA	Nasal swab	85.71%	100%	6.3 × 10^3^ TCID_50_/ml	Cross reactivity may occur with SARS‐CoV A low probability of cross reactivity with HKU1, Mycobacterium tuberculosis, and Pneumocystis jirovecii (PJP)
40	Nano‐Ditech Corp.	Nano‐Check COVID‐19 Antigen Test	LF‐CGIA	Nasopharyngeal swab	90.32%	100%	2.8 × 10^6^ TCID_50_/ml	Cannot rule out the cross reactivity with Mycobacterium tuberculosis and human coronavirus HKU1
41	iHealth Labs, Inc.	iHealth COVID‐19 Antigen Rapid Test	LF‐CGIA	Nasal swab	94.3%	98.1%	20 × 10^3^ TCID_50_/ml	Cannot rule out the cross reactivity with Mycobacterium tuberculosis, Pneumocystis jirovecii (PJP), and human coronavirus HKU1 Highly likely to have cross reactivity with SARS‐CoV
42	SD Biosensor, Inc.	COVID‐19 At‐Home Test	LF‐CGIA	Nasal swab	95.3%	100%	1.4 × 10^3^ TCID_50_/ml	Cross reactivity with SARS virus
43	Siemens Healthineers	CLINITEST Rapid COVID‐19 Antigen Self‐Test	LF‐CGIA	Nasal swab	86.5%	99.3%	7.0 × 10^3^ TCID_50_/ml	Cross reactivity with SARS virus
44	iHealth Labs, Inc.	iHealth COVID‐19 Antigen Rapid Test Pro	LF‐CGIA	Nasal swab	88.2%	100%	20 × 10^3^ TCID_50_/ml	Cannot rule out the cross reactivity with Pneumocystis jirovecii (PJP) and human coronavirus HKU1 Likely to have cross reactivity with SARS‐CoV
45	Siemens Healthcare Diagnostics, Inc.	ADVIA Centaur SARS‐CoV‐2 Antigen (CoV2Ag)	CLIA	Nasal swab	85.1%	100%	31.2 TCID_50_/ml	Cannot rule out the cross reactivity with human coronavirus HKU1 May have cross reactivity with SARS‐CoV
46	Siemens Healthcare Diagnostics, Inc.	Atellica IM SARS‐CoV‐2 Antigen (CoV2Ag)	CLIA	Nasal swab	85.1%	100%	31.2 TCID_50_/ml	Cannot rule out the cross reactivity with human coronavirus HKU1 May have cross reactivity with SARS‐CoV
47	Maxim Biomedical, Inc.	MaximBio ClearDetect COVID‐19 Antigen Home Test	LF‐CGIA	Nasal swab	86.9%	98.9%	750 TCID_50_/ml	Cannot rule out the cross reactivity with Mycobacterium tuberculosis, Pneumocystis jirovecii (PJP), and human coronavirus HKU1
48	PHASE Scientific International, Ltd.	INDICAID COVID‐19 Rapid Antigen At‐Home Test	LF‐CGIA	Nasal swab	81.7%	99.4%	2.8 × 10^3^ TCID_50_/ml	Cannot rule out the cross reactivity with Mycobacterium tuberculosis, Pneumocystis jirovecii (PJP), human coronavirus HKU1, and SARS‐CoV

Abbreviations: BAW, bulk acoustic wave biosensor; CLIA, chemiluminescence immunoassay; LF‐CGIA, lateral flow colloidal gold immunochromatographic assay; LF‐IFA, lateral flow immunofluorescence assay; MESIA, magnetic force‐assisted electrochemical sandwich immunoassay; MIFA, microfluidic immunofluorescence assay.

### 2.1 Lateral flow colloidal gold immunochromatographic assay (LF‐CGIA)

LF‐CGIA is a rapid and qualitative method for the determination of the presence of SARS‐CoV‐2 N protein in human respiratory samples including nasopharyngeal swab specimens. A sandwich technology is generally employed to test N protein in the LF‐CGIA. In brief, two kinds of monoclonal/polyclonal antibodies against the N protein of SARS‐CoV‐2 are immobilized on the testing line of the test strip and labeled with colloidal gold. When the lateral flow sample contains the N protein, colloidal gold‐labeled anti‐N protein antibodies bind to the N protein in the sample to form an antigen–antibody complex. This complex is then captured by anti‐N protein immobilized on the test line and a visible line appears on the membrane. A positive or negative result is indicated by a colored line appearing on the test region.^21^

Many research teams have evaluated LF‐CGIA for the SARS‐CoV‐2 N antigen, which showed that the sensitivity of LF‐CGIA differs from nearly 13% to 62%, like Daniela Basso has claimed the sensitivity could be 13% while Zehra Kipritci has reported the sensitivity could reach 61.8%, yet the specificity remained rather high, almost near 100% in most experiments^22^, ^23^ (Table 3). Nonetheless, the sensitivity was around 50% in most groups, such as in a real‐world comparison study in Florida, a total of 18,457 individuals were tested for the SARS‐CoV‐2 N antigen via LF‐CGIA and RT‐PCR assays for the SARS‐CoV‐2 RNA simultaneously. The positive percent agreement for the LF‐CGIA using the RT‐PCR comparator was only 49.2%. Even in symptomatic individuals, the positive percent agreement was just 51.9%.^24^ In another pairing study, 3419 specimens were included to test the SARS‐CoV‐2 N antigen. Compared with RT‐PCR assay, the LF‐CGIA had a sensitivity of 64.2% for specimens from symptomatic persons and 35.8% for those from asymptomatic persons, with almost 100% specificity in specimens from both groups.^25^

**TABLE 3:** Clinical evaluation of different assays for SARS‐CoV‐2 N protein antigen detection from included researches

First author	Assays	Name of the kits	Targets	Sample type	Groups (subgroups)	Sensitivity (%)	Specificity
Zehra Kipritci^23^	LF‐CGIA	SGA V‐Chek	N protein	NPS	–	61.80	100.00%
Lao‐Tzu Allan‐Blitz^24^	LF‐CGIA	Abbott BinaxNOW COVID‐19 antigen (Ag) card	N protein	NPS	Overall	49.20	98.80%
				NPS	Symptomatic	51.90	98.60%
Jessica L. Prince‐Guerra^25^	LF‐CGIA	Abbott BinaxNOW COVID‐19 Ag Card	N protein	NS	Symptomatic	64.20	100.00%
				NS	Asymptomatic	35.80	99.80%
				NS	Symptomatic (positive viral culture)	92.60	Not reported
				NS	Asymptomatic (positive viral culture)	78.60	Not reported
Nathalie Van der Moeren^26^	LF‐CGIA	BD Veritor System for Rapid Detection of SARS‐CoV‐2 (VRD)	N protein	NS	Overall	78.90	Not reported
				NS	7 days after symptoms onset	89.40	Not reported
				NS	Ct value <30	93.00	Not reported
Evangelos Terpos^27^	LF‐CGIA	COVID‐19 antigen detection kit (colloidal gold) manufactured by Zhuhai Lituo Biotechnology	N protein	NPS	CT PCR ≤25	100.00	99.59%
				NPS	CT PCR ≤33	99.00	99.59%
				NPS	CT PCR ≤40	89.47	99.59%
				NS	CT PCR ≤25	100.00	100.00%
				NS	CT PCR ≤33	96.12	100.00%
				NS	CT PCR ≤37	91.74	100.00%
Ilaria Baccani^30^	LF‐IFA	STANDARDTM F COVID‐19Ag FIA	N protein	NPS	STANDARD^TM^ F	35.71	100.00%
		AFIAS COVID‐19 Ag		NPS	AFIAS	37.50	100.00%
	CLIA	LUMIPULSE SARS‐CoV‐2 Ag kit x	N protein	NPS	Lumipulse^®^ G	87.88	95.83%
Rebecca L Smith^31^	LF‐IFA	Quidel SARS Sofia antigen fluorescent immunoassay (FIA)	N protein	NS and saliva	Positive viral culture	90	Not reported
Lisa J. Krüger^32^	MIFA	LumiraDx™ nucleocapsid (N) antigen protein	N protein	NS	Overall	82.19	99.35%
				NS	Heidelberg	84.62	99.29%
				NS	Berlin	80.25	99.48%
				NS	0‐7days	86.44	99.34%
				NS	8‐14days	53.85	100.00%
				NS	Symptomatic	82.48	99.14%
				NS	Asymptomatic	77.78	99.62%
Niko Kohmer^33^	LF‐IFA	RIDA^®^QUICK SARS‐CoV‐2 Antigen	Uncertain	NPS	R‐Biopharm	39.20	96.20%
	LF‐IFA	SARS‐CoV‐2 Rapid Antigen Test	N protein	NPS	Roche	43.20	100.00%
	LF‐IFA	NADAL^®^ COVID‐19 Ag Test	Uncertain	NPS	Nal von Minden GmbH	24.30	100.00%
	MIFA	LumiraDx™ nucleocapsid (N) antigen protein	N protein	NPS	LumiraDx GmbH	50.00	100.00%
Alana F Ogata^34^	ELISA	Single Molecule Array (Simoa)	S protein and N protein	NPS	Plasma	64.06	Not reported
Daniela Basso^22^	CLIA	LUMIPULSE SARS‐CoV‐2 Ag kit	N protein	NPS	Lumipulse^®^ G	81.60	93.80%
	LF‐CGIA	ESPLINE rapid test	Uncertain	NPS	Espline	48.00	100.00%
	LF‐CGIA	PanbioTM COVID‐19 Ag Rapid Test	N protein	NPS	Abbott	66.00	99.00%
	CLIA	LUMIPULSE SARS‐CoV‐2 Ag kit	N protein	Saliva	Lumipulse G	41.30	98.60%
	LF‐CGIA	ESPLINE rapid test	Uncertain	Saliva	Espline	13.00	Not reported
	LF‐CGIA	PanbioTM COVID‐19 Ag Rapid Test	N protein	Saliva	Abbott		
Gian Luca Salvagno^36^	CLIA	DiaSorin LIAISON	N protein	NPS	82 TCID_50_/ml	78.00	73.00%
Yosuke Hirotsu^37^	CLIA	LUMIPULSE SARS‐CoV‐2 Ag kit	N protein	NPS	–	55.20	99.60%
Qiaoling Deng^38^	CLIA	YHLO	N protein	Serum	Week 1	76.27	98.78%
				Serum	Week 2	62.50	Not reported

Abbreviations: CLIA, chemiluminescence immunoassay; ELISA, enzyme‐linked immunoabsorbent assay; LF‐CGIA, lateral flow colloidal gold immunochromatographic assay; LF‐IFA, lateral flow fluorescence immunoassays; MIFA, microfluidic immunofluorescence assay; NPS, nasopharyngeal swabs; NS, nasal swab.

A study about LF‐CGIA sensitivity stratified by PCR‐positive cycle threshold (Ct) Ct value and time since symptom onset showed that the overall sensitivity was 78.9%, whereas for specimen obtained within 7 days after symptom onset and for specimen with a Ct value of <30, the sensitivity was 89.4% and 93.0%, respectively.^26^ Another study showed that LF‐CGIA for the SARS‐CoV‐2 N antigen had a sensitivity of 100%, 99%, 89.47%, a specificity of 99.59%, 99.59%, 99.59%, and an accuracy of 99.68%, 99.42%, 96.37% in nasopharyngeal samples, when the RT‐PCR positive Ct values were ≤25, ≤33, and ≤40, respectively.^27^ When it comes to nasal swabs, the RT‐PCR positive Ct values were ≤25, ≤33, ≤37, and the LF‐CGIA sensitivity was 100%, 96.12%, 91.74%, separately, while the specificity was entirely 100%, and the accuracy was 98.78%, 98.87%, 97.49%, respectively. In addition, in specimens positive for viral culture, LF‐CGIA had a sensitivity of 92.6% for symptomatic and 78.6% for asymptomatic individuals.^25^

### 2.2 Lateral flow immunofluorescence assay (LF‐IFA)

The principle of LF‐IFA is similar to that of LF‐CGIA except the anti‐N protein antibodies labeled with fluorescein rather than colloidal gold.^28^ Accordingly, test results are identified using fluorescence intensity analyzer device rather than naked eyes. Improved LF‐IFA methods, such as microfluidic immunofluorescence assay (MF‐IFA), can achieve timed and quantitative immune response in the channel. Using biochip as the reaction channel, it can accurately control the uniform and orderly flow of microfluid and ensure the regular, orderly, and thorough immune reaction process. A positive or negative result is indicated by fluorescent signal on the test region.

A total of 1098 nasal swabs were tested for the SARS‐CoV‐2 N antigens via LF‐IFA. Of them, 871 were collected from asymptomatic participants, whereas the others were collected from symptomatic participants. LF‐IFA had a sensitivity of 41.2% and a specificity of 98.4% in swabs from asymptomatic participants, whereas in those from symptomatic participants, LF‐IFA test performance was improved (sensitivity = 80.0%; specificity = 98.9%).^29^ In another study, Ilaria Baccani and colleagues evaluated the performance of two LF‐IFAs. It was shown that they had a sensitivity of 35.7% and 37.5%, respectively, with an equally high specificity of 100%. These two assays had a sensitivity of 100% for samples with PCR‐positive Ct value of ≤25, whereas in samples with PCR‐positive Ct value of >30, the sensitivity decreased to 0.0%.^30^ In a longitudinal study of 43 adults newly infected with SARS‐CoV‐2, daily screening using LF‐IFA for the SARS‐CoV‐2 N antigen can achieve approximately 90% sensitivity for individuals when they are viral culture positive.^31^

Using MF‐IFA for the SARS‐CoV‐2 N antigens, Lisa et al. found that 120 of 146 PCR‐positive cases were detected to be positive, which showed that the MF‐IFA had a sensitivity of 82.19% and a specificity of 99.35%. In terms of the PCR‐positive Ct value, with the increasing PCR positive Ct value, the sensitivity declined from 92.63% to 41.67%.^32^ Niko Kohmer also evaluated a MF‐IFA using 100 clinical samples, and the sensitivity and specificity were 82.4% and 77.4%. Moreover, for the potentially infectious samples (≥10^6^ copies/ml), the MF‐IFA was found to have a sensitivity of 100%.^33^ It was also mentioned that sensitivity was elevated in individuals with a viral load of over log10^7^ copies/ml.^32^

### 2.3 Enzyme‐linked immunoabsorbent assay (ELISA)

ELISA is a qualitative or semi‐quantitative method for the determination of the SARS‐CoV‐2 N protein in human respiratory samples and plasma specimens. A sandwich technology is also employed to test N protein antigen in the ELISA. Usually, anti‐N protein antibody coats on the surface of microwells, then sample and enzyme‐labeled anti‐N protein detector antibody are mixed in a microwells. The N protein molecules presented in the sample are captured by the immobilized anti‐N protein, and subsequently labeled with enzyme. After washing clearly, a substrate of enzyme is added into microwells for color generation. The N protein concentration is positively correlated to color intensity.

Ogata and colleagues used ELISA to test SARS‐CoV‐2 N antigens and found it was detectable in 64.1% plasma from COVID‐19 positive patients. In these patients, full antigen clearance in plasma was observed a mean ± 95% CI of 5 ± 1 days after seroconversion, and nasopharyngeal RT‐PCR tests reported positive results for 15 ± 5 days after viral‐antigen clearance.^34^ In another study in Germany, ELISA was employed to test the SARS‐CoV‐2 N antigens in 107 PCR positive and 303 PCR‐negative respiratory swabs from asymptomatic and symptomatic patients as well as clinical isolates EU1 (B.1.117), variant of concern (VOC) Alpha (B.1.1.7) or Beta (B.1.351), and the sensitivity and specificity were 17.8% and 99.7%, while the calculated area under the curves (AUCs) was 0.65. In addition, ELISA is able to detect the SARS‐CoV‐2 N antigen of VOCs Alpha and Beta.^35^

### 2.4 Chemiluminescence immunoassay (CLIA) technology

CLIA is a high throughput and automatic method for qualitative or quantitative determination of the N protein of SARS‐CoV‐2 in samples collected and processed through the indicated preanalytical procedure. A direct two‐step sandwich CLIA is generally designed for the determination of the N protein of SARS‐CoV‐2. Specific polyclonal/monoclonal antibodies against the N protein are used for coating magnetic particles and linked to a chemiluminescence reagent. During the first incubation, the N protein antigen present in samples binds to anti‐N antibody on the magnetic particles. During the second incubation, chemiluminescence reagent antibody conjugate reacts with the N protein antigen already bound to the solid‐phase materials. After the second incubation, the unbound material is removed with washing. Subsequently, the starter reagents are added, and a flash chemiluminescence reaction is thus induced. The light signal reflecting the amount of SARS‐CoV‐2 is measured by a photomultiplier in relative light units.^36^

Daniela Basso et al. enrolled 234 patients for analyzing the clinical performance of a CLIA assay for SARS‐CoV‐2 N antigen, and found that it was highly accurate in distinguishing SARS‐CoV‐2 RNA‐positive and RNA‐negative nasopharyngeal swab with 81.6% sensitivity, 93.8% specificity, and 93.7% diagnostic accuracy.^22^ Ilaria Baccani and colleagues also evaluated the clinical performance of another CLIA assay. A total of 201 nasopharyngeal swabs were enrolled, including 33 from SARS‐CoV‐2 RNA‐positive and 168 from SARS‐CoV‐2 RNA‐negative patients. Results showed the CLIA assay had a sensitivity of 87.9% and a specificity of 95.8%, and appeared positive in almost all nasopharyngeal swabs with a Ct value ≤35 (92.6%), and 3 of 5 samples with a Ct value >35.^30^ Gian Luca Salvagno and his team recruited 421 patients for quantitation of the SARS‐CoV‐2 N antigen in nasal or nasopharyngeal swabs. Of them, 301 were tested for SARS‐CoV‐2 RNA positive.^36^ The median values in SARS‐CoV‐2 RNA positive samples was 94.8 TCID_50_/ml compared to 78.2 TCID_50_/ml in those testing negative, whilst that in samples associated with high infectivity risk was 3819.1 TCID_50_/ml compared to 82.0 TCID_50_/ml in those with lower infectivity risk. In the SARS‐CoV‐2 RNA positive samples, the Spearman's correlation analysis showed that the SARS‐CoV‐2 RNA N antigen levels were negative correlated to Ct values of the *E* (*r* = −0.85; *p* < 0.001) and *S* gene (*r* = −0.84; *p* < 0.001). The optimal cut‐off value for sample positivity was found to be 82 TCID_50_/ml, which resulting in 78% sensitivity, 73% specificity, and 77% diagnostic accuracy, whilst the optimal cut‐off value for high infective risk was 106 TCID_50_/ml, and the sensitivity, specificity, and diagnostic accuracy were 94%, 96%, and 95%, respectively.^36^ Yosuke Hirotsu tested 313 nasopharyngeal swabs using a RT‐PCR assay for SARS‐CoV‐2 RNA and a CLIA for SARS‐CoV‐2 N antigen. The median N antigen levels of the PCR positive and negative samples were 1.57 and 0.27 pg/ml (*p* < 0.05), and a positive correlation (*R* ^2^ = 0.768) was observed between the SARS‐CoV‐2 N antigen level and the viral load. The CLIA assay exhibited 55.2% sensitivity and 99.6% specificity, with a 91.4% overall concordance rate with RT‐PCR assay. The concordance rate gradually declined with decreasing viral load (100% concordance for samples with >100 copies/test, 60% for samples with 10–100 copies/test, 33% for samples with 1–10 copies/test, and 26% for samples with <1 copies/test).^37^ A retrospective study performed by Qiaoling Deng and colleagues to determine the SARS‐CoV‐2 N protein antigen levels by CLIA in 914 serum samples, including 309 collected from currently infected COVID‐19 patients and 48 from recovered ones. It was found group week 1 (0–7 days after COVID‐19 onset) had the highest level of serum SARS‐CoV‐2 N protein (15.02 COI), following by group week 2 (7–14 days after onset) (6.49 COI). In the first week, the sensitivity and specificity of serologic N protein antigen testing was 76.27% and 98.78%, respectively.^38^

### 2.5 Bulk Acoustic Wave (BAW) biosensor‐based immunoassay

An automated Bulk Acoustic Wave (BAW) biosensor‐based product from Qorvo Biotechnologies is an integrated system of instrument and reagent cartridges using immunoassay principles for the qualitative detection of the N antigens from SARS‐CoV‐2 in direct anterior nasal swab (NS) specimens.^39^ The instrument moves fluid from the sample port and various reagents from the cartridge carousel across the biosensor contained within the cartridge. On the surface of the biosensor an enzyme‐enhanced immune reaction takes place. Anti‐N protein antibody on the resonator surface captures the specific antigens to SARS‐CoV‐2. An enzyme‐conjugated anti‐N antibody binds to the immobilized SARS‐CoV‐2 antigens. The reaction causes a change in resonance frequency which is detected by the instrument. Results are then reported in Arbitrary Units/ml (AU/ml) and designated as “positive” or “negative” based on a set cut‐off value.

The manufactures collected prospectively 89 nasal swabs from 89 patients suspected of SARS‐CoV‐2 infection within 6 days from onset of symptoms, and found the sensitivity and specificity was 89.4% and 100.0% comparing to RT‐PCR, respectively, and moreover, the lowest limit of detection for this system was determined to be 200 TCID_50_/ml. To date, no other study reports to evaluate the clinical performance of this system.^39^

## 3 DISCUSSION

In this review, five immunoassays for detecting the SARS‐CoV‐2 N antigen, including LF‐CGIA, LF‐IFA, ELISA, CLIA, and BAW biosensor‐based immunoassay are introduced. Through the comparison among various immunoassays, it is apparent that the sensitivity exists significant difference. Table 3 has compiled the clinical evaluation of different assays for SARS‐CoV‐2 N protein antigen detection from the included researches. Using LF‐CGIA, the overall sensitivity varies from nearly 13%–62% in nasal or nasopharyngeal swabs and most of them are about 50%, whereas using CLIA ranges from 55.2% to 87.9%. One probable reason for significant difference is that there is a certain difference in the lowest limit of detection among various immunoassays. Another important reason is the obvious difference in constituent ratio of study participants. Supporting the former speculation, previous stratified studies showed that SARS‐CoV‐2 N antigen immunoassay sensitivity declined with the decreasing viral load in swabs and the increasing time after COVID‐19 symptom onset.^34^ In addition, SARS‐CoV‐2 N antigen was more detectable in swabs from symptomatic individuals than in those from asymptomatic individuals, and moreover, in SARS‐CoV‐2 culture positive swabs, approximately 90% of them were tested to be positive for SARS‐CoV‐2 N antigen. What's more, different types of variants could also have an impact on the sensitivity. The newly detected variant Omicron may reduce the sensitivity of antigen diagnostic tests resulting from its multiple mutations according to FDA, but it still lacks enough data and the experiment is ongoing.^40^ Due to the significant difference of sensitivity, it is necessary to validate or evaluate their clinical performances before starting the clinical laboratory, and then choosing the optimal immunoassay.

Although the optimal immunoassay has been chosen, how to interpret test result becomes extremely important due to low sensitivity. As shown in previous studies, the sensitivity of SARS‐CoV‐2 N antigen tests could drop to 13% in some occasions, and most of them were about 50%, which indicates that there are lots of false negative results. This may result from the comparatively high limit of detection of certain assay, so the low viral load of the sample could not reach the lowest limit of detection. Therefore, the negative results should be treated as presumptive if patients have one or more COVID‐19 symptoms and may be confirmed with an assay which has a lower limit of detection such as the RT‐PCR. As a result, for the asymptomatic patients whose viral load may be relatively low, N antigen tests cooperate with RT‐PCR results could be more reliable than only adopting N antigen tests. On the contrary, most of SARS‐CoV‐2 N immunoassays were reported to have a specificity of >95%, suggesting positive results generally indicate the presence of SARS‐CoV‐2 N antigen in samples. However, because the SARS‐CoV‐2 N protein closely resembles the SARS‐CoV N protein, nearly all the manufacturers of SARS‐CoV‐2 N protein immunoassay kits for EUA claim that the assays may have cross reactivity with SARS‐CoV and other viruses. Therefore, a positive result does not rule out other viral infections or coinfection with other bacteria, but cross reaction slightly occurs.

In addition to taking sensitivity and specificity into consideration, there are still some factors which may affect the usage of these immunoassays. First, LF‐CGIA and LF‐IFA are easy to use and have the ability to report test results rapidly on site, which could avoid the utilization of huge instruments. The turnaround time of SARS‐CoV‐2 N protein immunoassay is about half an hour, which is shorter than that of classical real‐time PCR assay (4 ~ 6 h). Places like the airport, custom, and harbor could utilize the lateral flow assays because they do not have enough space to hold a huge facility to have a high throughput and automatic determination of the N protein or construct a laboratory for RT‐PCR tests, and these places require the test results as soon as possible to avoid congestion. Thus, these places could utilize LF‐CGIA and LF‐IFA, and further diagnosis could be implemented during the quarantine through molecular assay in pursuit of precise results. As for some of the authorized self‐test kits for SARS‐CoV‐2 N antigen detection mentioned above, it is convenient for the suspected people to utilize lateral flow assays to test themselves in their homes during the quarantine. There is no need for doctors to visit each home to take specimens, which avoids the possibility of transmission, and the test results could be seen within 10–20 min and could be easily interpreted by themselves. Second, in the hospital, there is a laboratory for specific instruments to have a high throughput and automatic determination of the N protein or nucleic acid of SARS‐CoV‐2. Furthermore, hospitals would have a place to segregate the suspected people. Therefore, it is appropriate to have those time‐consuming but more precise assays including CLIA and RT‐PCR in the hospital.

Moreover, through hierarchical analysis, it was found that in the earlier stage of the disease, the higher viral load the sample contains, the easier the virus could be cultured successfully, ending up in a higher sensitivity and specificity of the N antigen detection.^6^, ^31^, ^38^ The analysis demonstrates that the transmissibility is positively related to the presence of N antigen detection. A patient with positive N antigen may be highly contagious, therefore, SARS‐CoV‐2 N antigen tests contribute to seeking out those infected people rapidly and dividing them from the uninfected people timely. They can be beneficial in congregate settings, such as workplace, school, or prison.^6^ These places could take antigen detection into consideration. If many workers, students, or prisoners share the same symptoms and one of them has already been diagnosed with COVID‐19, it is urgent for all the worker, students, or prisoners to take the SARS‐CoV‐2 N protein detection and they should be separated immediately, which could rapidly pick out those infected people and segregate those lucky dogs who may not be infected with COVID‐19.^41^ On the contrary, during the recovery period of COVID‐19, antigen detection may be reliable in predicting the clearance of virus due to the sensitivity correlation with viral load, which could be applied to shortening the period of recovery isolation stage. However, this opinion still needs further clinical trials because it is only proved by a novel SARS‐CoV‐2 human challenge model.^42^

## 4 CONCLUSION

In a nutshell, compared to the RT‐PCR and antibody detection, antigen detection has its unique advantages and we should make full use of it. Compared to RT‐PCR, it has shorter turnaround time and is free of instruments and experienced stuff, while compared to the antibody detection, it would not be influenced by the past infection and vaccination. It truly exists some drawbacks, but with time going by, its development and modification will benefit its utilization and broaden its usage, which contributes to finding out those infected people with rapidity and segregating them from the uninfected people.

## AUTHOR CONTRIBUTIONS

XL and MX performed the data analyses and wrote the manuscript. YL and XG contributed to the conception of the study. QD and YL contributed to the manuscript revision. All authors read and approved the submitted version.

## CONFLICT OF INTEREST

The authors declare no conflicts of interest.

## Data Availability

The authors confirm that the data supporting the findings of this study are available within the article.
